# Risk of breast cancer in men in relation to weight change: A national case‐control study in England and Wales

**DOI:** 10.1002/ijc.33938

**Published:** 2022-02-03

**Authors:** Anthony J. Swerdlow, Cydney Bruce, Rosie Cooke, Penny Coulson, Minouk J. Schoemaker, Michael E. Jones

**Affiliations:** ^1^ Division of Genetics and Epidemiology The Institute of Cancer Research London UK; ^2^ Division of Breast Cancer Research The Institute of Cancer Research London UK; ^3^ Nottingham Clinical Trials Unit, School of Medicine University of Nottingham Nottingham UK; ^4^ Oxford Cancer Centre Oxford University Hospitals NHS Foundation Trust Oxford UK

**Keywords:** breast cancer, men, weight change

## Abstract

Breast cancer is uncommon in men and knowledge about its causation limited. Obesity is a risk factor but there has been no investigation of whether weight change is an independent risk factor, as it is in women. In a national case‐control study, 1998 men with breast cancer incident in England and Wales during 2005 to 2017 and 1597 male controls were interviewed about risk factors for breast cancer including anthropometric factors at several ages. Relative risks of breast cancer in relation to changes in body mass index (BMI) and waist/height ratios at these ages were obtained by logistic regression modelling. There were significant trends of increasing breast cancer risk with increase in BMI from age 20 to 40 (odds ratio [OR] 1.11 [95% confidence interval (CI) 1.05‐1.17] per 2 kg/m^2^ increase in BMI; *P* < .001), and from age 40 to 60 (OR 1.12 [1.04‐1.20]; *P* = .003), and with increase in self‐reported adiposity compared to peers at age 11 to BMI compared with peers at age 20 (OR 1.19 [1.09‐1.30]; *P* < .001). Increase in waist/height ratio from age 20 to 5 years before diagnosis was also highly significantly associated with risk (OR 1.13 [1.08‐1.19]; *P* < .001). The associations with increases in BMI and waist/height ratio were significant independently of each other and of BMI or waist/height ratio at the start of the period of change analysed, and effects were similar for invasive and in situ tumours separately. Increases in BMI and abdominal obesity are each risk factors for breast cancer in men, independently of obesity per se. These associations might relate to increasing oestrogen levels with weight gain, but this needs investigation.

## Abstract 2

What's new?

Although obesity is an established risk factor for breast cancer in men, there have been no investigations of whether weight change is an independent risk factor, as it is in women. In an interview case‐control study including 1998 cases and 1597 controls, authors found that increase in body mass index and in abdominal obesity are each significant risk factors for breast cancer in men, independent of obesity per se.

**Abbreviations**
BMI: body mass index
CI: confidence interval
ER: oestrogen receptor
HER‐2: human epidermal growth factor receptor‐2
IGF‐1: insulin‐like growth factor‐1
OR: odds ratio

## 1 INTRODUCTION

Breast cancer occurs infrequently in men, about 1% of the incidence rate in women. Knowledge about its aetiology is sparse and has been investigated in relatively small studies with limited variables.^1^, ^2^, ^3^, ^4^, ^5^, ^6^ Raised risk in relation to obesity has been reported in several studies^1^, ^2^, ^3^, ^4^, ^5^, ^6^, ^7^, ^8^, ^9^ (Swerdlow et al, in press), and we have shown that this applies to both invasive and in situ tumours and is stronger for human epidermal growth factor receptor 2 (HER‐2) positive than HER‐2 negative tumours (Swerdlow et al, in press), for reasons unknown. We have also found that waist/height ratio is, independently, a risk factor (Swerdlow et al, in press). There have been no analyses, however, of risks in relation to changes in weight or waist size in men.

In postmenopausal women, the female age group most hormonally similar to men, increase in weight has been shown to be a risk factor for breast cancer,^10^, ^11^, ^12^, ^13^, ^14^, ^15^, ^16^, ^17^, ^18^ and decrease in weight has been shown in some studies to be associated with diminished risk,^13^, ^14^, ^17^, ^18^, ^19^ at least in women not taking exogenous hormones.

We have carried out a national interview‐based case‐control study of breast cancers incident in men in England and Wales since 2005, a far larger investigation than any previously. Here we analyse data from our study for risk of breast cancer, and subdivisions of this cancer, in relation to changes in both overall obesity and abdominal obesity.

## 2 MATERIALS AND METHODS

We conducted a population‐based case‐control study in which the potential cases were male residents of England and Wales diagnosed with breast cancer, invasive or in situ, in these countries during 1 January 2005 to 31 August 2017 at ages <80 years. These cases were identified comprehensively from population‐based cancer registries providing nationwide coverage and by notification to us by clinicians.

The study controls were derived from two sources, which enabled comparison between them to assess potential bias. One source was male non‐blood relatives of the cases—we asked each case whether we could approach their close non‐blood male relatives, and if so we selected one (or more) to interview based on approximate frequency matching (not pair matching) in strata by age and geographic region (in practice 1998 cases and 1597 controls). The other source was ‘Generations Study controls’—husbands of women taking part in the Generations cohort study,^20^ whom we approached via their wives, again based on frequency matching for the cases overall. All analyses presented in this article gave results in the same direction, and generally very similar, using each control group separately, so the two groups were combined for the tables presented.

The potential cases were approached via their consultants to take part, and potential controls by mail. If they agreed, they were then visited by a trained research nurse who interviewed them using a structured questionnaire and took a blood (or failing that saliva) sample for genetic analyses. The questionnaire asked about a wide range of demographic and potential risk factors, including height at age 20 years, weight at ages 20, 40 and 60 years, height and adiposity compared with peers at age 11 (much thinner, a little thinner, about the same, a little fatter, much fatter) and waist circumference based on trouser size at age 20 and 5 years before interview (prediagnostic time‐points that we thought might be memorable for this more‐difficult to remember variable).

We analysed the data by standard methods for case‐control studies,^21^ calculating odds ratios (referred to below as relative risks) adjusted for ‘index’ age (see below), marital status, socioeconomic status, region of residence and year of interview using a logistic regression model. To provide an ‘index age’ for controls, equivalent to the age at diagnosis for cases, to use in the above adjustment, we calculated for each calendar year of interview, the mean interval from cancer diagnosis to interview for cases and then subtracted this interval from the age at interview of each control who had been interviewed in the same year. To compare degree of adiposity at age 11, which was based on reported comparison with peers, and at age 20, which was based on body mass index (BMI), we categorised adiposity at age 11 according to percentile cut‐offs derived from the distribution in the control group, and then applied the same percentiles to the controls at age 20 to provide BMI cut‐offs at that older age. Thus, for instance, 69 (4.3%) controls reported that they had been in the lowest adiposity category, ‘much thinner’, at age 11 and 458 (28.7%) reported that they had been in the next category, ‘a little thinner’ at age 11. We therefore demarcated the lowest BMI category at age 20 as the bottom 4.3% of control BMIs at that age, and the next category as the control BMIs at age 20 from 4.3% up to 33.0%, and so on; that is, the lowest category comprised the same percentage of controls at age 11 (4.3%) as at age 20 (4.3%), and the same was true for each subsequent category.

We analysed linear trends in relative risks across exposure levels as a continuous variable^21^ and present *z* scores to compare the strengths of these trends for exposures that were measured on different scales, and case‐case analyses to test for interaction. All analyses were conducted with STATA 16.0.^22^

The study interviewers recorded at the end of the interview, their opinion of the reliability of the participant's responses. We then conducted sensitivity analyses in which we excluded subjects whose quality of responses were rated ‘not well’ or ‘very poorly’. We also conducted sensitivity analyses excluding from analysis men with known Klinefelter syndrome; excluding unmarried men rather than adjusting for marital status (because one control group, the Generations Study controls, was entirely married); and adjusting for factors that are known or possible risk factors for breast cancer in men, although not clear confounders: chest radiotherapy, family history of breast cancer, testicular conditions and use of exogenous oestrogens or androgens.

## 3 RESULTS

In total, 3187 men diagnosed with breast cancer at ages under 80 years in England and Wales during the study period were reported to us by the cancer registries or consultants across the country. Four hundred and twenty seven (13.4%) of these had died before we could contact them, for 21 (0.7%) the consultant did not participate or we could not ascertain the consultant, 28 (0.9%) were deemed by their consultant as unsuitable to approach, 6 (0.2%) had emigrated and 707 (22.2%) declined or did not reply to the invitation to take part. This left 1998 (62.7%) who were interviewed and were the study cases. Most (71%) were aged 60 or older, 92% of tumours were invasive and the great majority (99% of known) were oestrogen receptor (ER) positive (Table 1). The 1998 cases were similar to the 1189 non‐participant eligible men with breast cancer in age (averages 63.9 and 65.1 years, respectively), diagnosis date (65.7% and 63.6% from 2010 onward, respectively), percent of tumours invasive rather than in situ (92.9% and 94.0%, respectively), but not socioeconomic (ACORN) score (42.1% vs 25.7% from groups 1 plus 2). Similarly participant controls were more often higher social class than men invited to be controls who did not participate (57.9% vs 52.9%, respectively).

**TABLE 1:** Descriptive characteristics of cases and controls

Characteristic	Cases		Generations Study husband controls		Non‐blood relative controls		All controls	
	No.	%	No.	%	No.	%	No.	%
Index age (years)^a^								
<40	47	2.4	8	0.8	76	12.4	84	5.3
40‐49	159	8.0	13	1.3	135	22.1	148	9.3
50‐59	385	19.2	118	12.0	165	26.9	283	17.7
60‐69	729	36.5	564	57.3	162	26.4	726	45.5
70‐79	678	33.9	281	28.6	75	12.2	356	22.3
Region of residence								
North	375	18.8	172	17.5	91	14.5	263	16.5
North‐West	368	18.4	193	19.6	98	15.6	291	18.2
Mids and E (incl Wales)	379	18.9	154	15.7	131	20.9	285	17.8
London and SE	465	23.3	270	27.4	159	25.3	429	26.9
South West	411	20.6	195	19.8	149	23.7	344	21.6
Socio‐economic group (ACORN)^b^								
1 (highest)	710	35.5	594	60.4	271	44.2	865	54.2
2	120	6.0	35	3.5	25	4.1	60	3.8
3	633	32.2	279	28.4	204	33.3	483	30.2
4	318	15.9	57	5.8	77	12.5	134	8.4
5 (lowest)	183	9.2	12	1.2	32	5.2	44	2.7
Uncategorised^c^	24	1.2	7	0.7	4	0.7	11	0.7
Year of interview								
2007‐2009	447	22.4	214	21.7	229	37.4	443	27.7
2010‐2014	807	40.4	533	54.2	245	40.0	778	48.7
2015‐2020	744	37.2	237	24.1	139	22.7	376	23.6
Invasiveness of breast cancer								
Invasive	1838	92.0						
In situ	160	8.0						
Oestrogen receptor status of breast cancer								
+ve	1844	92.3						
−ve	28	1.4						
Not known^d^	126	6.3						
Total	1998	100	984	100	613	100	1597	100

^a^ Age at diagnosis of cases; equivalent age for controls (see Section 2).

^b^ Acorn score based on postcode of residence (CACI, 2019).

^c^ Geographic areas not covered by Acorn (Isle of Man, Channel Islands), and residence in an institution or other non‐domestic situation.

^d^ 97.2% of invasive; 53.1% of in situ.

Of 828 men approached to be non‐blood relative controls, 613 (74%) participated in the study and of 1109 men approached to be Generations Study controls, 984 (89%) participated.

Risk of breast cancer increased significantly with increase in BMI from age 20 to 40 (OR 1.11 [1.05‐1.17]; *P* < .001) and from age 40 to 60 (OR 1.12 [1.04‐1.20]; *P* = .003) (Table 2). There was also a highly significant association of rising risk with increasing degree of adiposity from age 11 to 20 (OR 1.19 [1.09‐1.30]; *P* < .001; see Section 2). The trends with change in adiposity/BMI in Table 2 were slightly stronger when adjustment was added for initial adiposity/BMI category at the start of the age period analysed (Table S1).

**TABLE 2:** Risk of breast cancer in men in relation to change in relative adiposity/body mass index from ages 11 to 20, 20 to 40 and 40 to 60 years

	Cases no.	Controls no.	OR^a^	95% CI	*P*	
Change in relative adiposity, age 11 to 20^b^						
<0	366	357	0.85	0.70‐1.03	.09	
0	865	736	1.00	Baseline		
1	475	350	1.18	0.98‐1.42	.08	
≥2	102	53	1.65	1.14‐2.38	.008	
Not known	190	101	1.48	1.11‐1.96	.006	
Trend^b^			1.19	1.09‐1.30	<.001	*z* = 3.77
Change in BMI, age 20 to 40						
<0	65	44	1.28	0.82‐2.01	.27	
0	390	312	1.00	Baseline		
1‐2	539	527	0.87	0.71‐1.07	.19	
3‐4	392	309	1.09	0.87‐1.37	.45	
≥5	366	214	1.35	1.06‐1.73	.02	
Not known	199	107	1.40	1.04‐1.90	.03	
Not yet age 40	47	84				
Trend^c^			1.11	1.05‐1.17	<.001	*z* = 3.77
Change in BMI, age 40 to 60						
<0	135	115	0.94	0.69‐1.29	.74	
0	438	362	1.00	Baseline		
1‐2	397	352	1.02	0.82‐1.27	.84	
3‐4	189	144	1.12	0.84‐1.49	.43	
≥5	156	64	1.95	1.37‐2.79	<.001	
Not known	92	45	1.35	0.88‐2.07	.17	
Not yet age 60	591	515				
Trend^c^			1.12	1.04‐1.20	.003	*z* = 3.01

Abbreviations: BMI, body mass index, kg/m^2^; CI, confidence interval; OR, odds ratio.

^a^ Adjusted for age, socioeconomic status (Acorn score [CACI, 2019]), region of residence, year of interview and marital status.

^b^ Adiposity at age 11 in five categories of comparison with peers. BMI at age 20 was split into categories with the percentile cut‐offs between the categories matching those for controls at age 11. The change values represent moves between these categories, rather than a numerical change in BMI. Linear trend excludes ‘Not known’ category and is per unit increase in category.

^c^ Linear trend excludes ‘Not known’ category, and is per 2 unit increase in BMI (ie, per 1 category in the table).

Change in waist/height ratio from age 20 to 5 years before interview was also related to risk (Table 3): there was a strong gradient of increasing risk with greater waist/height ratio gain (OR 1.13 [1.08‐1.19]; *P* < .001), with a stronger effect (*z* = 4.91) than for any of the above BMI changes (*z* = 3.77, 3.77 and 3.01 for changes from ages 11 to 20, 20 to 40 and 40 to 60 respectively). Adjustment for initial waist/height ratio increased this association (Table S2).

**TABLE 3:** Risk of breast cancer in men in relation to change in waist/height ratio from age 20 to 5 years before interview

	Cases no.	Controls no.	OR^a^	95% CI	*P*	
Change in waist/height ratio, age 20 to 5 years before interview						
<0.00	25	22	0.99	0.50‐1.95	.97	
0.00	150	143	1.00	Baseline		
0.01‐0.03	360	350	1.12	0.83‐1.51	.45	
0.04‐0.06	394	389	1.12	0.83‐1.50	.46	
0.07‐0.09	260	224	1.25	0.90‐1.71	.18	
≥0.10	371	192	1.78	1.29‐2.43	<.001	
Not known	438	277	1.53	1.07‐2.20	.02	
Trend^b^			1.13	1.08‐1.19	<.001	*z* = 4.91

Abbreviations: CI, confidence interval; OR, odds ratio.

^a^ Adjusted for age, socioeconomic status (Acorn score [CACI, 2019]), region of residence, year of interview and marital status.

^b^ Linear trend, excluding ‘Not known’ category, per 0.03 units increase in waist to height ratio (ie, per 1 category increase in the table).

When we adjusted BMI change for waist/height ratio change, and vice versa, taking BMI change from age 20 to 60 as the most comparable to the waist/height ratio time points, change in waist/height ratio (OR 1.11 [1.04‐1.19]; *P* = .002), and BMI (OR 1.07 [1.01‐1.13]; *P* = .03) remained independently significant (Table 4).

**TABLE 4:** Risk of breast cancer in men in relation to change in body mass index (BMI) between ages 20 and 60 years, and to change in waist/height ratio between age 20 and 5 years before interview, with and without mutual adjustment

	Cases no.	Control no.	Mutually unadjusted^a^				Mutually adjusted^b^			
			OR^a^	95% CI	*P*		OR^b^	95% CI	*P*	
Change in BMI										
<0	70	44	1.37	0.84‐2.25	.21		1.54	0.91‐2.59	.10	
0	156	131	1.00	Baseline			1.00	Baseline		
1‐2	264	266	0.95	0.69‐1.30	.75		0.89	0.64‐1.23	.50	
3‐4	252	253	0.96	0.70‐1.32	.81		0.86	0.62‐1.21	.38	
≥5	514	318	1.47	1.09‐1.98	.01		1.21	0.87‐1.69	.25	
Not known	151	70	1.90	1.27‐2.84	.002		1.70	1.11‐2.62	.02	
Not yet age 60	591	515								
Trend^c^			1.11	1.05‐1.16	<.001	*z* = 3.82	1.07	1.01‐1.13	.03	*z* = 2.12
Change in waist/height ratio^d^										
<0.00	15	15	0.92	0.39‐2.16	.85		0.68	0.28‐1.65	.39	
0.00	97	95	1.00	Baseline			1.00	Baseline		
0.01‐0.03	223	231	1.10	0.76‐1.60	.61		1.18	0.81‐1.74	.39	
0.04‐0.06	278	265	1.27	0.88‐1.82	.20		1.33	0.90‐1.96	.15	
0.07‐0.09	180	167	1.31	0.89‐1.93	.17		1.31	0.86‐2.00	.20	
≥0.10	289	150	1.86	1.27‐2.73	.001		1.76	1.15‐2.68	.009	
Not known	325	159	1.62	1.06‐2.49	.03		1.38	0.87‐2.20	.17	
Not yet age 60	591	515								
Trend^c^			1.13	1.07‐1.20	<.001	*z* = 4.23	1.11	1.04‐1.19	.002	*z* = 3.04

Abbreviations: BMI, body mass index, kg/m^2^; CI, confidence interval; OR, odds ratio.

^a^ Adjusted for age, socioeconomic status (Acorn score [CACI, 2019]), region of residence, year of interview and marital status.

^b^ Additionally adjusted for change in waist/height ratio or for change in BMI, as appropriate.

^c^ Linear trend excluding ‘Not known’ category, per 2 unit increase in BMI or 0.03 increase in waist/height ratio (ie, per 1 category in the table).

^d^ Restricted to men age ≥60, in order that they can have a value for the adjustment variable.

In analyses separately for invasive and in situ cancers (Tables S3 and S4), odds ratios for each of these categories were similar to those for breast cancer overall, but significance levels were far greater for invasive tumours, which were the great majority, and sometimes not significant for in situ tumours, which were only 8% of the total. Likewise, in separate analyses for HER‐2 negative and positive tumours (Tables S5 and S6), odds ratios were not significantly different between them, but trends were highly significant for the former, which were the great majority, and mainly not significant for the latter, which were <10% of the total (Table S5). There were too few ER negative tumours to analyse by ER status.

Sensitivity analyses excluding men with Klinefelter syndrome, or excluding non‐married men, or excluding men whose responses were rated by the interviewer as ‘not well’ or ‘very poorly’, or adjusting for the potential confounders described in the Section 2, made no material difference to the Results (not in table). Addition of adjustment for smoking and alcohol consumption, as possible confounders, although there is no established evidence for them as risk factors for breast cancer in men, made no material difference to the results: for instance the linear trend odds ratios in Tables 2, 3, 4 were unchanged or at most altered by 0.01.

## 4 DISCUSSION

Obesity is an important risk factor for breast cancer in men^1^, ^2^, ^3^, ^4^, ^5^, ^6^, ^7^, ^8^, ^9^ (Swerdlow et al, in press), but we have found here that gain in BMI, and more so gain in waist/height ratio, are also independent risk factors. There have not to our knowledge been any previous analyses of male breast cancer risk in relation to weight change, but in postmenopausal women weight gain has been associated with increased risk in most^10^, ^11^, ^12^, ^13^, ^14^, ^15^, ^16^, ^17^, ^18^ but not all^19^, ^23^ studies. As in the men in our study, the association in women has been found independent of initial weight.^11^, ^12^, ^13^, ^14^, ^15^, ^16^, ^17^ There do not appear to be previous analyses of the effect of change in waist circumference on breast cancer risk in men or in women. Our results suggest that this change has a greater effect than does change in BMI. This accords with our previous finding that breast cancer risk in men relates more closely to waist circumference than it does to BMI, reinforcing the importance of abdominal fat to risk (Swerdlow et al, in press).

A likely mechanism for the effect of weight change on risk is changes in sex hormone levels. Half or more of circulating oestradiol in men derives from aromatisation of testosterone in adipose tissue,^24^ and obese men have higher oestrogen levels^25^, ^26^, ^27^, ^28^, ^29^, ^30^ and oestrogen production levels^25^ than non‐obese men. Decreases in weight of obese men through bariatric surgery^31^ or a fasting programme^32^ lead to decreased serum levels of oestradiol, so it seems likely that increased weight would lead to increased oestradiol levels.

We are not aware of any studies of oestradiol levels in relation to changes in waist circumference, but free circulating oestradiol levels in men are associated with waist circumference,^28^, ^29^ so it is plausible that increases in waist circumference might lead to increased oestradiol levels. The stronger independent effect of waist circumference change than BMI change might reflect the greater metabolic activity of abdominal visceral adipocytes than other adipocytes.^33^

In postmenopausal women, IGF‐1 levels as well as oestrogen levels have been posited as potential links between obesity and breast cancer risk.^34^ However, this seems unlikely to explain our findings on changes in obesity in men because reduction in visceral obesity has been found to be associated with increased IGF‐1 levels in men.^35^ In postmenopausal women, insulin resistance is associated with breast cancer risk,^36^ so might be relevant in men, although we know of no evidence for it. In women insulin resistance is particularly associated with visceral obesity, but this is not so in men.^37^

In women testosterone levels are associated with subsequent breast cancer risk,^38^ testosterone levels in men are inversely related to BMI and central obesity,^29^, ^30^, ^39^ so, although we cannot find any evidence on the effect of changes in male obesity on testosterone levels, it seems unlikely that testosterone level changes could explain our findings.

Our study has the strengths of large numbers of cases (the previous individual studies of male breast cancer have included less than 12% of the number of cases^2^ and none have analysed changes in weight/BMI or waist circumference), national systematic ascertainment of cases and exposure information from personal interview. However, because, like almost all of the literature, it is a case‐control study (a cohort study of this size would not be practical), it is potentially at risk of the biases that can arise with this design.^21^ The inclusion of two control sources, with similar results from each, and with high response rates, reduces the likelihood of the presence of control selection bias. The exposure measures used in the study were self‐reported, but such reports have been shown to be well correlated with measured weight and waist circumference in men.^40^

In summary, this analysis from a large case‐control study shows that increase in BMI and independently increase in waist/height ratio are associated with raised risk of breast cancer in men, with waist/height ratio being the stronger association. Effects via circulating oestrogen levels are a potential mechanism.

## CONFLICT OF INTEREST

The authors declare no conflicts of interest.

## ETHICS STATEMENT

The South East Research Ethics Committee approved the study (07/MRE01/1). The participants gave individual written consent at recruitment.

## Supporting information


**Table S1** Risk of breast cancer in men in relation to change in relative fatness/body mass index from ages 11 to 20, 20 to 40 and 40 to 60 years, adjusted for baseline fatness/BMI
**Table S2** Risk of breast cancer in men in relation to change in waist/height ratio from age 20 to 5 years before interview, adjusted for waist/height ratio at age 20
**Table S3** Risk of invasive, and of in situ, breast cancer in men in relation to change in relative fatness/body mass index from ages 11 to 20, 20 to 40 and 40 to 60 years
**Table S4** Risk of invasive, and of in situ, breast cancer in men in relation to change in waist/height ratio from age 20 to 5 years before interview
**Table S5** Risk of breast cancer in men in relation to change in relative fatness/body mass index from ages 11 to 20, 20 to 40 and 40 to 60 years, by HER‐2 expression status
**Table S6** Risk of breast cancer in men in relation to change in waist/height ratio from age 20 to 5 years before interview, by HER‐2 expression status

## Data Availability

Data that are minimally required to replicate the outcomes of the study will be made available upon request to the corresponding author (https://www.icr.ac.uk).
