# Supplementary material for: Risk of breast cancer in men in relation to weight change: A national case‐control study in England and Wales
Source: Int J Cancer. 2022 Feb 3;150(11):1804–11. doi: 10.1002/ijc.33938 (PMC9303449; doi:10.1002/ijc.33938)
Supplement: Supplementary file 1 — Table S1 Risk of breast cancer in men in relation to change in relative fatness/body mass index from ages 11 to 20, 20 to 40 and 40 to 60 years, adjusted for baseline fatness/BMI Table S2 Risk of breast cancer in men in relation to change in waist/height ratio from age 20 to 5 years before interview, adjusted for waist/height ratio at age 20 Table S3 Risk of invasive, and of in situ, breast cancer in men in relation to change in relative fatness/body mass index from ages 11 to 20, 20 to 40 and 40 to 60 years Table S4 Risk of invasive, and of in situ, breast cancer in men in relation to change in waist/height ratio from age 20 to 5 years before interview Table S5 Risk of breast cancer in men in relation to change in relative fatness/body mass index from ages 11 to 20, 20 to 40 and 40 to 60 years, by HER‐2 expression status Table S6 Risk of breast cancer in men in relation to change in waist/height ratio from age 20 to 5 years before interview, by HER‐2 expression status [file IJC-150-1804-s001.pdf]

Supplementary Material for

Risk of breast cancer in men in relation to weight change: a national case-control study in England and Wales

Anthony J Swerdlow, Cydney Bruce, Rosie Cooke, Penny Coulson, Minouk J Schoemaker, Michael E Jones

Supplementary tables 1-6

Suppl. Table 1 Risk of breast cancer in men in relation to change in relative fatness/body mass index from ages 11-20, 20-40 and 40-60 years, adjusted for baseline fatness/BMI

	Cases No.	Controls No.	OR[a]	95% CI	P	
**Change in relative fatnesss, age 11 to 20[b]**						
<0	366	357	0.81	0.66-0.99	0.04	
0	865	736	1.00	Baseline	.	
1	475	350	1.22	1.00-1.48	0.05	
≥2	102	53	1.74	1.15-2.63	0.008	
Not known	178	101	1.41	1.06-1.88	0.02	
Trend[b]			1.23	1.10-1.37	< 0.001	z = 3.58
**Change in BMI, age 20 to 40**						
<0	65	44	1.12	0.71-1.77	0.63	
0	390	312	1.00	Baseline	.	
1-2	539	527	0.89	0.72-1.10	0.28	
3-4	392	309	1.13	0.90-1.43	0.29	
≥5	366	214	1.42	1.10-1.82	0.007	
Not known	199	107	1.20	0.52-2.77	0.67	
Not yet age 40	47	84				
Trend[c]			1.13	1.07-1.20	< 0.001	z = 4.37
**Change in BMI, age 40 to 60**						
<0	135	115	0.80	0.58-1.12	0.19	
0	438	362	1.00	Baseline	.	
1-2	397	352	1.02	0.82-1.27	0.89	
3-4	189	144	1.07	0.81-1.43	0.63	
≥5	156	64	1.89	1.32-2.71	<0.001	
Not known	92	45	1.95	0.66-5.77	0.23	
Not yet age 60	591	515				
Trend[c]			1.14	1.06-1.23	< 0.001	z = 3.57

OR = Odds ratio; CI = Confidence interval; BMI = Body mass index, Kg/m^2

[a] Adjusted for age, socioeconomic status (Acorn score (CACI, 2019)), region of residence, year of interview and marital status.

[b] Fatness at age 11 in 5 categories of comparison with peers. BMI at age 20 was split into categories with the percentile cut-offs between the categories matching those for controls at age 11. The change values represent moves between these categories, rather than a numerical change in BMI. Linear trend excludes 'Not known' category and is per unit increase in category.

[c] Linear trend excludes 'Not known' category, and is per 2 unit increase in BMI (i.e. per 1 category in the table).

Suppl. Table 2 Risk of breast cancer in men in relation to change in waist/height ratio from age 20 to 5 years before interview, adjusted for waist/height ratio at age 20

	Cases No.	Controls No.	OR[a]	95% CI	P	
**Change in waist/height ratio, age 20 to 5 years before interview**						
<0.00	25	22	0.83	0.41-1.67	0.60	
0.00	150	143	1.00	Baseline	.	
0.01-0.03	360	350	1.17	0.87-1.58	0.30	
0.04-0.06	394	389	1.20	0.89-1.62	0.24	
0.07-0.09	260	224	1.35	0.97-1.87	0.08	
≥0.10	371	192	1.95	1.40-2.72	<0.001	
Not known	438	277	1.26	0.71-2.25	0.43	
Trend[b]			1.16	1.10-1.23	<0.001	z = 5.55

OR = Odds ratio
CI = Confidence interval
[a] Adjusted for age, socioeconomic status (Acorn score (CACI, 2019)), region of residence, year of interview and marital status.
[b] Linear trend, excluding 'Not known' category, per 0.03 units increase in waist to height ratio (i.e. per 1 category increase in the table).

Suppl. Table 3 Risk of invasive, and of in situ, breast cancer in men in relation to change in relative fatness/body mass index from ages 11-20, 20-40, 40-60 years

	**Invasive**						**In situ**					
	**Cases No.**	**Controls No.**	**OR[a]**	**95% CI**	**P**		**Cases No.**	**Controls No.**	**OR[a]**	**95% CI**	**P**	
**Change in relative fatness, age 11 to 20[b]**												
<0	343	357	0.87	0.71-1.05	0.15		23	357	0.67	0.41-1.11	0.12	
0	732	736	1.00	Baseline	.		72	736	1.00	Baseline	.	
1	475	350	1.18	0.98-1.43	0.08		38	350	1.07	0.70-1.65	0.75	
≥2	91	53	1.61	1.11-2.36	0.01		11	53	2.16	1.04-4.49	0.04	
Not known	174	101	1.48	1.10-1.97	0.009		16	101	1.51	0.82-2.81	0.19	
Trend[b]			1.18	1.07-1.29	< 0.001	z = 3.51			1.29	1.04-1.61	0.02	z = 2.35
P interaction in trends invasive/in situ[d]	0.34											
**Change in BMI, age 20 to 40**												
<0	58	44	1.22	0.77-1.94	0.39		7	44	2.31	0.90-5.93	0.08	
0	366	312	1.00	Baseline	.		24	312	1.00	Baseline	.	
1-2	494	527	0.86	0.70-1.06	0.17		45	527	1.04	0.61-1.79	0.89	
3-4	360	309	1.08	0.85-1.36	0.54		32	309	1.35	0.76-2.41	0.30	
≥5	337	214	1.35	1.05-1.73	0.02		29	214	1.40	0.76-2.59	0.28	
Not known	183	107	1.40	1.03-1.90	0.03		16	107	1.59	0.78-3.27	0.21	
Not yet age 40	40	84					7	84				
Trend[c]			1.11	1.05-1.18	< 0.001	z =3.81			1.06	0.92-1.21	0.42	z = 0.81
P interaction in trends invasive/in situ[d]	0.68											

OR = Odds ratio; CI = Confidence interval; BMI = Body mass index, Kg/m^2

[a] Adjusted for age, socioeconomic status (Acorn score (CACI, 2019)), region of residence, year of interview and marital status.

[b] Fatness at age 11 in 5 categories of comparison with peers. BMI at age 20 was split into categories with the percentile cut-offs between the categories matching those for controls at age 11. The change values represent moves between these categories, rather than a numerical change in BMI. Linear trend excludes 'Not known' category and is per unit increase in category.

[c] Linear trend excludes 'Not known' category, and is per 2 unit increase in BMI (i.e. per 1 category in the table).

[d] From case-case analyses.

(Contd.) Suppl. Table 3 Risk of invasive, and of in situ, breast cancer in men in relation to change in relative fatness/body mass index from ages 11-20, 20-40, 40-60 years

	**Invasive**						**In situ**					
	**Cases No.**	**Controls No.**	**OR[a]**	**95% CI**	**P**		**Cases No.**	**Controls No.**	**OR[a]**	**95% CI**	**P**	
**Change in BMI, age 40 to 60**												
<0	122	115	0.90	0.66-1.25	0.54		13	115	1.69	0.78-3.65	0.18	
0	415	362	1.00	Baseline	.		23	362	1.00	Baseline	.	
1-2	373	352	1.00	0.81-1.26	0.97		24	352	1.14	0.61-2.13	0.67	
3-4	177	144	1.10	0.83-1.47	0.52		12	144	1.40	0.65-3.00	0.39	
≥5	144	64	1.91	1.33-2.74	<0.001		12	64	3.09	1.38-6.93	0.006	
Not known	85	45	1.33	0.86-2.06	0.10		7	45	2.40	0.90-6.37	0.08	
Not yet age 60	522	515					69	515				
Trend[c]			1.10	1.02-1.18	0.01	z = 2.61			1.09	0.90-1.32	0.21	z = 0.89
P interaction in trends invasive/in situ[d]						0.65						

OR = Odds ratio; CI = Confidence interval; BMI = Body mass index, Kg/m^2

[a] Adjusted for age, socioeconomic status (Acorn score (CACI, 2019)), region of residence, year of interview and marital status.

[b] Fatness at age 11 in 5 categories of comparison with peers. BMI at age 20 was split into categories with the percentile cut-offs between the categories matching those for controls at age 11. The change values represent moves between these categories, rather than a numerical change in BMI. Linear trend excludes 'Not known' category and is per unit increase in category.

[c] Linear trend excludes 'Not known' category, and is per 2 unit increase in BMI (i.e. per 1 category in the table).

[d] From case-case analyses.

Suppl. Table 4 Risk of invasive, and of in situ, breast cancer in men in relation to change in waist/height ratio from age 20 to 5 years before interview

	Invasive						In situ					
	**Cases No.**	**Controls No.**	**OR[a]**	**95% CI**	**P**		**Cases No.**	**Controls No.**	**OR[a]**	**95% CI**	**P**	
**Change in waist/ height ratio, age 20 to 5 years before interview**												
<0.00	23	22	0.98	0.49-1.97	0.96		2	22	0.65	0.13-3.23	0.60	
0.00	131	143	1.00	Baseline	.		19	143	1.00	Baseline	.	
0.01-0.03	329	350	1.20	0.88-1.64	0.25		31	350	0.67	0.36-1.27	0.22	
0.04-0.06	367	389	1.21	0.89-1.65	0.22		27	389	0.53	0.28-1.01	0.06	
0.07-0.09	240	224	1.33	0.95-1.86	0.09		20	224	0.78	0.39-1.56	0.48	
≥0.10	336	192	1.83	1.32-2.54	<0.001		35	192	1.54	0.82-2.92	0.18	
Not known	412	277	1.72	1.18-2.49	0.005		26	277	0.67	0.29-1.58	0.36	
Trend[b]			1.13	1.08-1.19	< 0.001	z = 4.80			1.17	1.04-1.31	0.009	z = 2.61
P interaction in trends invasive/in situ[c]						0.57						

OR = Odds ratio
CI = Confidence interval
[a] Adjusted for age, socioeconomic status (Acorn score (CACI, 2019)), region of residence, year of interview and marital status.
[b] Linear trend, excluding 'Not known' category, per 0.03 units increase in waist to height ratio (i.e. per 1 category increase in the table).
[c] From case-case analyses.

Suppl. Table 5 Risk of breast cancer in men in relation to change in relative fatness/body mass index from ages 11-20, 20-40, 40-60 years, by HER-2 expression status

	**HER-2 Positive**						**HER-2 Negative**					
	**Cases No.**	**Controls No.**	**OR[a]**	**95% CI**	**P**		**Cases No.**	**Controls No.**	**OR[a]**	**95% CI**	**P**	
**Change in relative fatness, age 11 to 20[b]**												
<0	34	357	0.80	0.51-1.25	0.32		262	357	0.88	0.71-1.08	0.23	
0	89	736	1.00	Baseline	.		601	736	1.00	Baseline	.	
1	40	350	0.91	0.59-1.39	0.65		331	350	1.21	0.99-1.48	0.06	
≥2	13	53	2.45	1.22-4.91	0.01		70	53	1.67	1.12-2.49	0.01	
Not known	12	101	1.06	0.52-2.16	0.86		128	101	1.48	1.08-2.01	0.01	
Trend[b]			1.17	0.95-1.44	0.14	z = 1.49			1.19	1.08-1.31	< 0.001	z = 3.52
P interaction in trends HER-2 +ve/-ve[d]						0.84						
**Change in BMI, age 20 to 40**												
<0	7	44	1.58	0.61-4.09	0.35		48	44	1.24	0.77-2.00	0.38	
0	36	312	1.00	Baseline	.		288	312	1.00	Baseline	.	
1-2	54	527	0.96	0.59-1.57	0.89		356	527	0.80	0.64-1.01	0.06	
3-4	32	309	0.98	0.57-1.70	0.94		282	309	1.09	0.85-1.39	0.51	
≥5	41	214	1.85	1.08-3.18	0.03		250	214	1.26	0.97-1.65	0.09	
Not known	15	107	1.46	0.71-2.97	0.30		137	107	1.34	0.97-1.86	0.08	
Not yet age 40	3	84					31	84				
Trend[c]			1.18	1.04-1.34	0.01	z = 2.58			1.11	1.04-1.17	< 0.001	z = 3.37
P interaction in trends HER-2 +ve/-ve[d]						0.63						

OR = Odds ratio; CI = Confidence interval; BMI = Body mass index, Kg/m^2

[a] Adjusted for age, socioeconomic status (Acorn score (CACI, 2019)), region of residence, year of interview and marital status.

[b] Fatness at age 11 in 5 categories of comparison with peers. BMI at age 20 was split into categories with the percentile cut-offs between the categories matching those for controls at age 11. The change values represent moves between these categories, rather than a numerical change in BMI. Linear trend excludes 'Not known' category and is per unit increase in category.

[c] Linear trend excludes 'Not known' category, and is per 2 unit increase in BMI (i.e. per 1 category in the table).

[d] From case-case analyses.

(Contd.) Suppl. Table 5 Risk of breast cancer in men in relation to change in relative fatness/body mass index from ages 11-20, 20-40, 40-60 years, by HER-2 expression status

	HER-2 Positive						HER-2 Negative					
	Cases No.	Controls No.	OR[a]	95% CI	P		Cases No.	Controls No.	OR[a]	95% CI	P	
**Change in BMI, age 40 to 60**												
<0	15	115	0.96	0.48-1.90	0.90		91	115	0.92	0.65-1.30	0.63	
0	53	362	1.00	Baseline	.		304	362	1.00	Baseline	.	
1-2	26	352	0.62	0.36-1.06	0.08		290	352	1.05	0.83-1.33	0.68	
3-4	15	144	0.72	0.36-1.43	0.35		143	144	1.22	0.90-1.65	0.19	
≥5	16	64	2.12	1.05-4.27	0.04		113	64	2.05	1.41-2.99	<0.001	
Not known	4	45	0.94	0.31-2.82	0.91		65	45	1.40	0.88-2.22	0.15	
Not yet age 60	59	515					387	515				
Trend[c]			1.00	0.84-1.19	0.98	z = 0.03			1.15	1.06-1.24	< 0.001	z = 3.49
P interaction in trends HER-2 +ve/-ve[d]						0.12						

OR = Odds ratio; CI = Confidence interval; BMI = Body mass index, Kg/m^2

[a] Adjusted for age, socioeconomic status (Acorn score (CACI, 2019)), region of residence, year of interview and marital status.

[b] Fatness at age 11 in 5 categories of comparison with peers. BMI at age 20 was split into categories with the percentile cut-offs between the categories matching those for controls at age 11. The change values represent moves between these categories, rather than a numerical change in BMI. Linear trend excludes 'Not known' category and is per unit increase in category.

[c] Linear trend excludes 'Not known' category, and is per 2 unit increase in BMI (i.e. per 1 category in the table).

[d] From case-case analyses.

Suppl. Table 6 Risk of breast cancer in men in relation to change in waist/height ratio from age 20 to 5 years before interview, by HER-2 expression status

	HER-2 Positive						HER-2 Negative					
	**Cases No.**	**Controls No.**	**OR[a]**	**95% CI**	**P**		**Cases No.**	**Controls No.**	**OR[a]**	**95% CI**	**P**	
**Change in waist/ height ratio, age 20 to 5 years before interview**												
<0.00	4	22	1.27	0.36- 4.54	0.71		17	22	1.08	0.51- 2.28	0.85	
0.00	18	143	1.00	Baseline	.		100	143	1.00	Baseline	.	
0.01-0.03	34	350	0.75	0.39- 1.45	0.39		258	350	1.30	0.93- 1.81	0.12	
0.04-0.06	34	389	0.63	0.33- 1.22	0.17		306	389	1.36	0.98- 1.88	0.07	
0.07-0.09	24	224	0.72	0.35- 1.46	0.36		192	224	1.48	1.04- 2.11	0.03	
≥0.10	38	192	1.32	0.68- 2.54	0.41		261	192	1.93	1.36- 2.73	<0.001	
Not known	36	277	1.01	0.45- 2.26	0.98		258	277	1.87	1.26- 2.78	0.002	
Trend[b]			1.08	0.97-1.21	0.16	z = 1.41			1.14	1.08-1.20	< 0.001	z = 4.65
P interaction in trends HER-2 +ve/-ve[c]						0.35						

OR = Odds ratio
CI = Confidence interval
[a] Adjusted for age, socioeconomic status (Acorn score (CACI, 2019)), region of residence, year of interview and marital status.
[b] Linear trend, excluding 'Not known' category, per 0.03 units increase in waist to height ratio (i.e. per 1 category increase in the table).
[c] From case-case analyses.
